# Phytochemical profile, enzyme inhibition activity and molecular docking analysis of *Feijoa sellowiana* O. Berg

**DOI:** 10.1080/14756366.2021.1880397

**Published:** 2021-02-08

**Authors:** Fatema R. Saber, Rehab M. Ashour, Ali M. El-Halawany, Mohamad Fawzi Mahomoodally, Gunes Ak, Gokhan Zengin, Engy A. Mahrous

**Affiliations:** a Pharmacognosy Department, Faculty of Pharmacy, Cairo University, Cairo, Egypt; b Institute of Research and Development, Duy Tan University, Da Nang, Vietnam; c Faculty of Science, Department of Health Sciences, University of Mauritius, Réduit, Mauritius; d Department of Biology, Science Faculty, Selcuk University, Campus, Konya, Turkey

**Keywords:** Antioxidant, acetylcholinesterase, amylase, Feijoa sellowiana, glucosidase, molecular docking, Pineapple guava, tyrosinase

## Abstract

*Feijoa sellowiana* leaves and fruits have been investigated as a source of diverse bioactive metabolites. Extract and eight metabolites isolated from *F. sellowiana* leaves were evaluated for their enzymatic inhibitory activity against α-glucosidase, amylase, tyrosinase, acetylcholinestrerase and butyrylcholinesterase both *in vitro* and *in silico*. *Feijoa* leaves’ extract showed strong antioxidant activity and variable levels of inhibitions against target enzymes with a strong anti-tyrosinase activity (115.85 mg Kojic acid equivalent/g). Additionally, α-tocopherol emerged as a potent inhibitor of AChE and BChE (5.40 & 10.38 mmol galantamine equivalent/g, respectively). Which was further investigated through molecular docking and found to develop key enzymatic interactions in AChE and BChE active sites. Also, primetin showed good anti BChE (11.70 mmol galantamine equivalent/g) and anti-tyrosinase inhibition (90.06 mmol Kojic acid equivalent/g) which was also investigated by molecular docking studies.

**Highlights**
- Isolation of eight bioactive constituents from *Feijoa sellowiana* leaves.
- *In vitro* assays using different enzymatic drug targets were investigated.
- *In silico* study was performed to define compound interactions with target proteins.
- *Feijoa* leaf is an excellent source of anti-AChE and antityrosinase bioactives.

## Graphical Abstract

**Figure:**
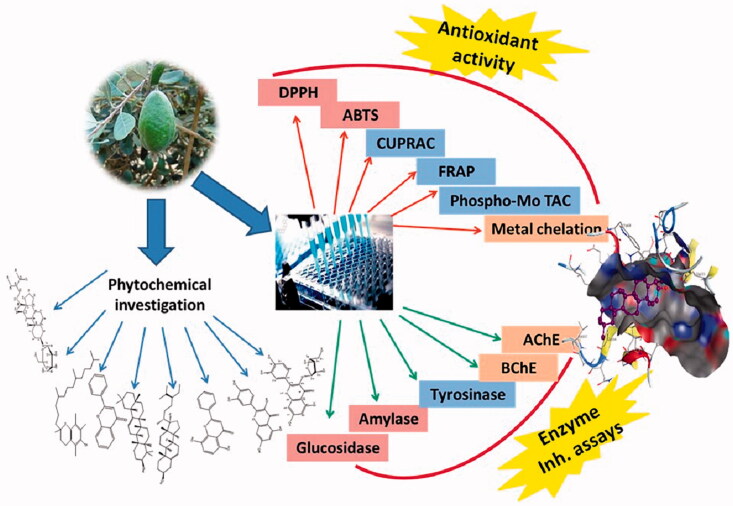

## 1. Introduction

*Acca sellowiana* O. Berg*. syn*. *Feijoa sellowiana* O. Berg. belongs to the Myrtaceae family. It is commonly known as pineapple guava or guavasteen due to its fruit’s characteristic aroma and close resemblance to guava, *Psidium guajava* L. of the same family^1^^,^^2^. The plant is native to South America but has been introduced to different climatic regions including the Mediterranean^1^. It is cultivated for its edible aromatic fruit and also, as ornamental trees. The introduction of *Feijoa* in different climates resulted in the availability of many varieties of the plant with different genotypes and chemical compositions^2^.

In its native countries, *Feijoa* fruits and flowers are used to treat certain conditions either internally in the treatment of common cold or externally for the treatment of ulcers and sores^1^^,^^2^. As infusion, the leaves are used to treat diarrhoea, dysentery and in the management of diabetes^3–5^. Several pharmacological studies have been conducted to validate these uses and explore additional biological activities of different plant organs especially its edible fruits^2^. Recent reports suggested a broad spectrum of biological activities including antifungal, anti-inflammatory, antioxidant, anti-diabetic and antitumor activities^6–9^.

The chemical constituents of different organs of *Feijoa* have also been investigated in earlier studies. Methyl, ethyl and (Z)-3-hexenyl benzoates have been identified as important key volatiles in feijoa fruits^10^^,^^11^. Aside from the essential oil, phenolic compounds were identified as the major class of secondary metabolites in *Feijoa*^4^^,^^12^. Chemical investigation of leaves, fruits and fruit peel showed the presence of many phenolic compounds including ferulic acid, ellagic acid, chlorogenic acid, catechin, quercetin and its glycosides as major secondary metabolites in different organs of *Feijoa*^12^^,^^13^.

As a part of our continuing effort to examine plants of Myrtaceae family for their bioactive principles^14^^,^^15^, the present study was designed to further investigate the chemical constituents of *Feijoa* leaves. Additionally, different *in vitro* assays (antioxidant and enzyme inhibition) were conducted to provide some additional insights and perspectives into the possible health benefits of *F. sellowiana* in management of different human diseases and possible mechanistic insights by performing the same assays for the major encompassed chemical constituents.

## 2. Materials and methods

### General procedures

Silica gel G 60 (E-Merck, Darmstadt, Germany). Silica gel H (E-Merck, Darmstadt, Germany). Thin-layer chromatography (TLC) was performed on silica gel GF254 pre-coated plates (Fluka, Steinheim, Germany). Solvent systems used for TLC development, were: S_1_: *n*-hexane-ethyl acetate (95:5 v/v), S_2_: *n*-hexane-ethyl acetate (85:15 v/v), S_3_: methylene chloride-methanol (95:5 v/v) + few drops formic acid and S_4_: methylene chloride-methanol (85:15 v/v) + few drops formic acid. The chromatograms were visualised by spraying with natural product/polyethylene glycol (NP/PEG) and *p*-anisaldehyde sulphuric spray reagents. Solvent used for extraction and fractionation are all of analytical grade.

Bruker AVIIIHD400 FT–NMR Spectrometer (400/3) instrument (Japan) was used for ^1^H and ^13 ^C-NMR analyses (^1^H-400 MHz and ^13 ^C-100 MHz) measurements and chemical shifts were given in *δ* value. TMS was used as internal standard.

### Plant material

Leaves of *Feijoa sellowiana* O. Berg were collected from Zohria botanic garden in May 2016. The plant was kindly identified by Mrs. Therese Labib, Botanical specialist and consultant at Orman and Qubba Botanic Gardens, Giza, Egypt. Voucher specimen of the plant material number (1.7.2019–2) was deposited at the herbarium of Pharmacognosy Department, Faculty of Pharmacy, Cairo University.

### Preparation of extract and pure compounds’ isolation

One kg of the powdered leaves was extracted by Soxhlet apparatus using methylene chloride: methanol mixture (80:20 v/v) as a solvent to yield 85 grams of the dried extract as previously adopted^15^. Thirty grams of the extract was fractionated using vacuum liquid chromatography packed with 300 g of silica gel H (17.5 × 5 cm) and eluted with petroleum ether 100%, and then increasing polarity by addition of ethyl acetate until 100% ethyl acetate. This was followed by addition of 1% increments of methanol (1–10% methanol in EtOAc). Fractions were collected, 200 ml each, and monitored by TLC. Similar fractions were pooled together to give four collective main fractions (I–IV).

Fraction I (5% EtOAc in *n*-hexane, 1.5 g) was subjected to three successive steps of column chromatography on silica gel G eluted with 5% ethyl acetate in *n*-hexane v/v to yield 80 mg of yellow oil (**F1**). Fraction II (10–20% EtOAc in *n*-hexane, 4 g) was re-chromatographed using silica gel G column eluted with 10% v/v ethyl acetate in *n*-hexane to yield 40 subfractions that were monitored using TLC. Similar subfractions were pooled together and concentrated to yield three collective subfractions 1–3. Subfraction 1 was subjected to reversed phase chromatography using C_18_-bonded silica gel eluted with methanol to give two compounds **F2** (white needles, 51 mg), and **F6** (white needles 40 mg). Meanwhile, subfractions 2 and 3 upon concentration and recrystallization gave two compounds **F3** (white needles, 23 mg) and **F7** (white needles, 30 mg), respectively.

Fraction III (50% EtOAc in n-hexane, 1 g) was subjected to gel filtration on Sephadex LH-20 column eluted with 100% methanol to give 27 mg of yellow powder (**F4**). Finally fraction IV (5% methanol in EtOAc, 3.7 g) was re-chromatographed on silica G column eluted with a gradient of methanol and methylene chloride up to 5% methanol v/v to yield two compounds **F8** (white powder, 20 mg) and **F5** (yellow powder, 35 mg).

### Total phenolic and total flavonoid content

Total phenolic content was determined using Folin–Ciocalteu method as described previously by Slinkard and Singleton^16^ and was calculated as gallic acid equivalent (GAE). On the other hand, total flavonoids content was determined by using AlCl_3_ method according to Uysal et al.^17^ and was expressed as rutin equivalent (RE).

### HPLC-PDA characterisation of feijoa extract

Chromatographic separation was achieved using Agilent1260 infinity II (Agilent technologies, Santa Clara, CA, USA) using C18-bonded RP LiChrospher®100 column (250 × 4.6 mm, 5 µm particle size). A binary gradient of solvent A (1% aqueous trifluroacetic acid TFA), solvent B (1% TFA in CAN) at flow rate of 1 ml/min was used for separation as follows: 95%A:5% B at 0 min, 15% A:85% B at 15 min, 0%A: 100%B at 18 min followed by isocratic elution at 100% B for 3 min followed by a return to starting condition at 25 min. UV absorbance of eluted compounds was recorded at *λ* 250, 280 and 325 nm, injection volume was set at 20 µL. Feijoa extract was dissolved in methanol (HPLC grade, Sigma, Aldrich, MO., USA) at 5 mg/mL, mixed and passed through 0.22 µm syringe filter. Serial dilutions of external standard quercetin (0.1–1 mg/mL), flavone (0.1–1 mg/mL) and avicularin (0.05–0.5 mg/mL) were similarly prepared and analysed under the same conditions. *Feijoa* extract was analysed in triplicate experiments and concentration of the three constituents was calculated using standard calibration curve as mean ± SD HPLC Chromatograms are provided as Supplementary data, Figure S1.

### Determination of antioxidant activity

Measurement of the antioxidant potential of the extract and its isolated compounds was done using several assay models previously described by Uysal et al^18^ including radical scavenging assays for ABTS^·^ (2,2′-azino-bis(3-ethylbenzothiazoline-6-sulphonic acid) and DPPH (2,2-diphenyl-1-picrylhydrazyl) radicals and different redox assays such as FRAP assay (ferric reducing antioxidant power) and CUPRAC assay (cupric reducing antioxidant capacity), in addition to, phosphomolybdenum total antioxidant capacity (TAC). Finally, metal chelating assay was also performed as metals are known catalyst for oxidation reactions. Trolox and EDTA (for chelating assay) were used as reference compounds.

### Determination of enzyme inhibitory properties

*Feijoa* extract and its isolated compounds were tested for possible enzyme inhibition activity against several drug targets of different human diseases. All the assays were done according to methods previously described by Uysal et al.^18^ and Sut et al.^19^. For α-glucosidase inhibition assay, PNPG (4-N-trophenyl-α-D-glucopyranoside, Sigma) was used as a substrate for α-glucosidase solution (*Saccharomyces cerevisiae*), while α-amylase inhibitory activity was measured by incubating test samples with α-amylase (ex-porcine pancreas, EC 3.2.1.1, Sigma) followed by addition of starch and iodine-potassium iodide solution for colour development. In both assays, acarbose was used as a reference drug and the results were calculated as mmol acarbose equivalent per gram of extract ACAE/g. Galantamine was used as a reference standard drug for acetylcholinesterase AChE (Electricell AChE, Type-VI-S, EC 3.1.1.7, Sigma) and butyrylcholinesterase BChE (horse serum BChE, EC 3.1.1.8, Sigma) inhibition assays with acetylthiocholine iodide and butylthiocholine chloride as substrates, respectively. Activity of test samples was expressed as galantamine equivalent per gram of extract GALE/g. Finally, antityrosinase activity was measured against mushroom tyrosinase using L-DOPA as a substrate and activity was calculated in reference to kojic acid as mmol kojic acid equivalent per gram KAE/g. All assays were performed in 96-well plates and run in triplicate for each tested sample.

### Molecular docking studies

Potential ligands were docked using Molecular Operating Environment MOE Version 2015.1 (Chemical Computing Group, Canada). Prior to docking procedures, potential ligands were compiled as mol files into a database and energy minimisation was performed using Amber 10:EHT. Enzymes were downloaded from Protein Data Bank (http://www.rcsb.org/) in complex with a known inhibitor, preferably the one used in the *in vitro* study when available, Table S1 (provided in the supplementary file). For each enzyme, hydrogen was added and 3 D protonation was performed using MOE specific function. The co-crystallised ligand was then removed and redocked in the active site using Triangle Matcher placement and London dG scoring. After successful redocking of the original ligand (RMSD value <1.5 Å), the same docking parameters were used for tested compounds in addition to another competitive inhibitor for each enzyme as illustrated in Table S1 to determine key interactions. Enzyme ligand interactions were visualised using the computing function of the program.

### Statistical analysis

All experiments were done in triplicate and were expressed as mean ± standard deviation. Statistical analysis was performed using one-way ANOVA followed by Tukey's test. Significant difference was set at (*p* < 0.05).

## 3. Results and discussion

### Phytoconstituents of *Feijoa* leaves extract

**Figure 1.**
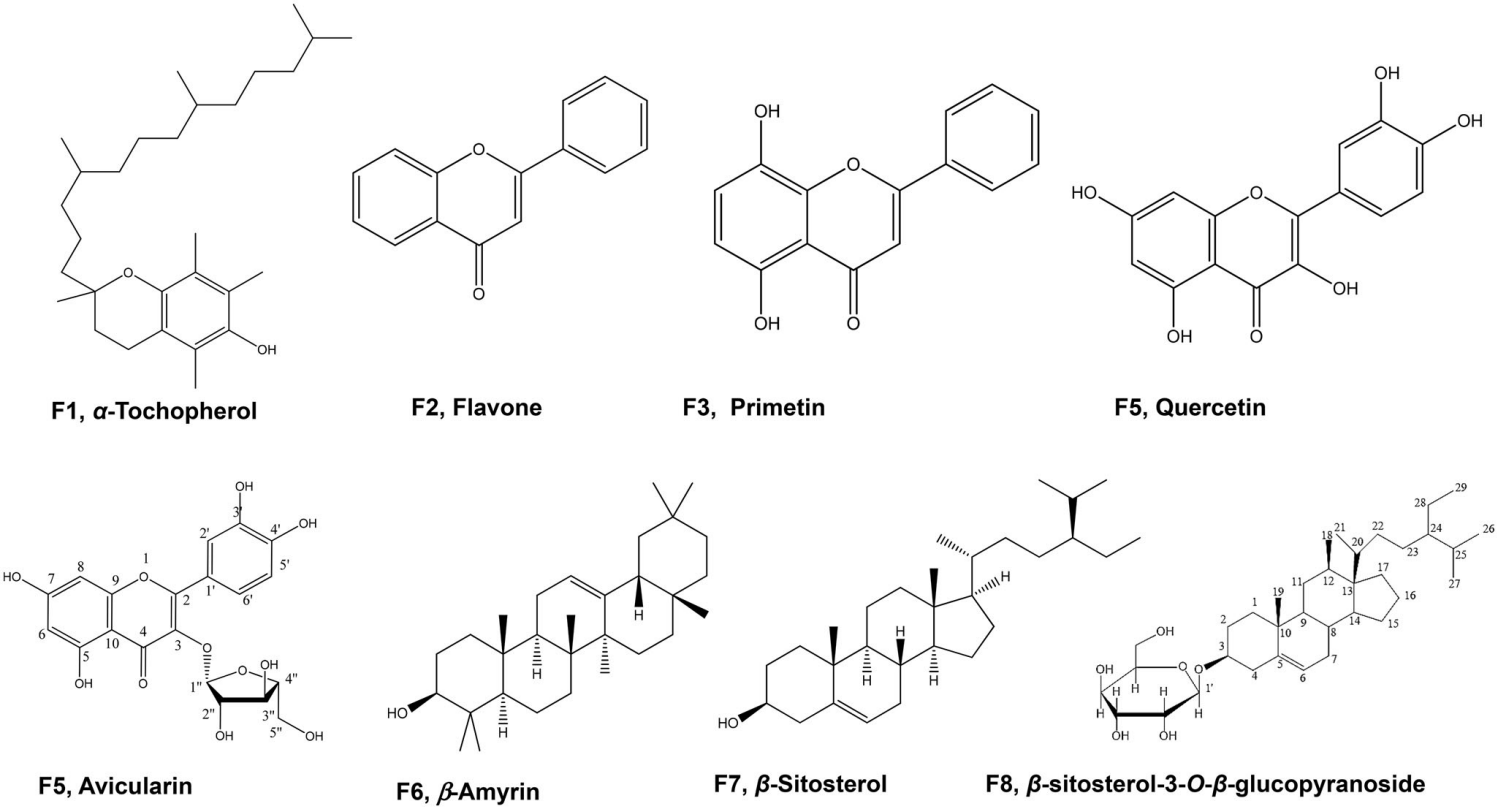
Structures of compounds isolated from *Feijoa sellowiana* leaves*’* extract.

*Feijoa* leaves extract was found to be rich in phenolic content as determined by the Folin–Ciocalteu method which estimated the phenolic content per gram of the extract at 128.87 ± 0.92 mg GAE. Additionally, relatively high flavonoid content was also reported for the extract at 71.37 ± 0.23 mg RE per gram of extract. This was supported by thorough phytochemical investigation of *Feijoa* extract which resulted in the isolation of several compounds including; α-tocopherol **F1** and four flavonoids, namely, flavone **F2**, primetin **F3**, quercetin **F4**, and its 3-*O-*arabinofuranoside glycoside (avicularin), **F5** (Figure 1). These compounds were identified based on co-chromatography with authentic compounds, as well as, their characteristic NMR spectra, being compared with the available literature^8^^,^^11^^,^^20^. NMR data are presented in Tables S2 and S3 (provided in the supplementary file). Among these compounds, avicularin has been previously identified as a major metabolite of *Feijoa* leaves^8^^,^^13^, while vitamin E (*α*-tocopherol), flavone and quercetin were previously reported in both leaf and fruit extracts^12^^,^^21^. Additionally, one triterpene, *β*-amyrin, **F6** and two phytosterols: *β*-sitosterol, **F7** and *β*-sitosterol glucoside**, F8** were isolated and identified based on their ^1^H-NMR and ^13 ^C-NMR and by comparison with the current available literature^21^^,^^22^.

Among isolated compounds, flavone was the most abundant as inferred from quantitative HPLC-UV results with concentration 74.38 ± 4.27 mg/g of extract followed by avicularin 4.33 ± 0.062 mg/g and quercetin 444.34 ± 83.15 µg/g.

### Antioxidant activity

It is widely accepted that oxidative stress is a prominent contributing factor in a number of serious health ailments including diabetes, Alzheimer's disease, multiple sclerosis among many^23^^,^^24^. Plants that are generally rich in phenolic compounds, have been widely recognised as powerful natural antioxidants such as green tea and pomegranate^23^^,^^24^. Also, some of the isolated compounds such as α-tocopherol and quercetin are well known for their antioxidant properties which have been reported in multiple studies^25^^,^^26^. The high phenolic content reported herein and from previous studies for *Feijoa* extract prompted us to further evaluate its antioxidant capacity using different assays that can highlight different aspects of the antioxidant capacity of the extract. In this regard, the radical scavenging activity, antioxidant capacity, metal chelating activity and reduction potential of the extract in addition to its isolated compounds were carried out using different models.

*Feijoa* extract showed good radical scavenging activity as compared to the standard drug Trolox at 90.58 and 113.80 mg TE/g extract in DPPH and ABTS^·^ assays, respectively. Similarly, *Feijoa* extract showed good antioxidant activity in all used assay model (Table 1). This strong activity is likely due to the presence of powerful antioxidant compounds *α*-tocopherol and quercetin and its glycoside avicularin as can be inferred from Table S4^27^^,^^28^ (provided in the supplementary file). The presence of powerful antioxidant compounds with wide range of lipid solubility including the lipid soluble α-tocopherol and the glycoside avicularin may afford good antioxidant activity of *Feijoa* extract in different biological systems^27^^,^^28^.

**Table 1.** Antioxidant activity of *Feijoa sellowiana* leaves’ extract.

Assay type	Results of *Feijoa* extract
DPPH^a^	90.58 ± 0.89
ABTS^a^	113.80 ± 0.02
FRAP^a^	102.58 ± 0.41
CUPRAC^a^	180.23 ± 0.44
Metal chelating activity^b^	21.21 ± 0.88
Phosphomolybdenum assay^a^	5.31 ± 0.13

Values are expressed as mean ± SD.

^a^mg Trolox equivalent/g, ^b^ mg EDTA equivalent/g.

### Antidiabetic activity

For potential anti-diabetic activity, inhibitory activity against *α-*glucosidase and pancreatic *α-*amylase enzymes was evaluated. These are two model enzymes used to evaluate *in vitro* antidiabetic activity through inhibition of the hydrolysis of dietary carbohydrate^29^. Inhibitors of both enzymes are effective treatment in reducing postprandial hyperglycaemia in diabetic patients and a known inhibitor for both enzymes, acarbose, is widely used as a standard treatment for type-II diabetes^30^.

*Fejioa* extract showed good *α-*glucosidase and *α-*amylase inhibitory activity at 1.06 and 1.52 mmol acarbose equivalent ACAE per gram of the extract, respectively, (Table 2). Among tested compounds, primetin showed the best activity against both enzymes, while other flavonoids quercetin and avicularin showed weak activity against amylase <1 mmol ACAE/g and were inactive against *α-*glucosidase. Flavonoids have been previously evaluated as α-glucosidase and α-amylase inhibitors by other investigators and the differential activity of flavonoids of different structures have been previously reported^29^^,^^31^. In a study of different flavonoids, substitution at position 3 of the flavone structure was found to reduce amylase inhibitory activity supporting the good amylase inhibitory activity observed for primetin, as compared to quercetin, (Table 2). It is worth mentioning that in a recent study on 44 flavonoids, that did not include primetin, quercetin was identified as the strongest α-glucosidase inhibitor^32^. Discrepancies in the literature in reporting inhibitory activity against α-glucosidases can be attributed to different experimental conditions including source of enzyme, incubation time, and enzyme concentration, among others^33^.

**Table 2.** Inhibitory activity of *Feijoa sellowiana* leaves’ extract and its isolated compounds using different assay models.

Samples	AChE (mg GALAE/g)	BChE (mg GALAE/g)	Amylase (mmol ACAE/g)	Glucosidase (mmol ACAE/g)	Tyrosinase (mg KAE/g)
Total extract	4.18 ± 0.37 ^cd^	2.55 ± 0.16^e^	1.06 ± 0.01^bc^	1.52 ± 0.01^d^	115.85 ± 2.55^b^
*α*-Tocopherol	4.92 ± 0.07^b^	10.38 ± 0.53^abc^	0.62 ± 0.02^f^	1.54 ± 0.01^d^	79.67 ± 0.83^de^
Flavone	na	10.20 ± 0.70^bc^	1.17 ± 0.07^b^	1.62 ± 0.01^ab^	77.31 ± 5.07^e^
Primetin	5.40 ± 0.05^a^	11.70 ± 0.10^a^	3.76 ± 0.13^a^	1.60 ± 0.01^bc^	90.06 ± 3.31^c^
Quercetin	4.44 ± 0.02^c^	11.08 ± 0.15^ab^	0.73 ± 0.09^ef^	na	163.05 ± 5.19^a^
Avicularin	3.80 ± 0.17^d^	na	0.73 ± 0.02^ef^	na	90.85 ± 0.87^c^
*β*-Amyrin	5.25 ± 0.06^ab^	na	0.87 ± 0.09^de^	1.62 ± 0.01^a^	90.65 ± 4.77^c^
*β*-Sitosterol	4.97 ± 0.07^b^	9.07 ± 0.37 ^cd^	0.56 ± 0.02^f^	1.61 ± 0.01^abc^	86.87 ± 2.02 ^cd^
*β-*Sitosterol glucoside	5.23 ± 0.03^ab^	8.40 ± 1.11^d^	0.96 ± 0.01 ^cd^	1.60 ± 0.01^c^	85.44 ± 0.93^cde^

Values are expressed as mean ± SD. GALAE: Galantamine equivalent; ACAE: Acarbose equivalent; KAE: Kojic acid equivalent; na: not active.

Different letters indicate significant differences in the samples (*p* < 0.05).

To gain insights into how the observed *in vitro* activity of *Feijoa* extract and its isolated compounds may be translated into therapeutic outcome, we used molecular docking to visualise ligand protein interactions of the isolated compounds in the active site of intestinal glucoamylase and pancreatic α-amylase; the two main α-glucosidases responsible for releasing glucose from dietary carbohydrate in the intestine^34^^,^^35^.

**Figure 2.**
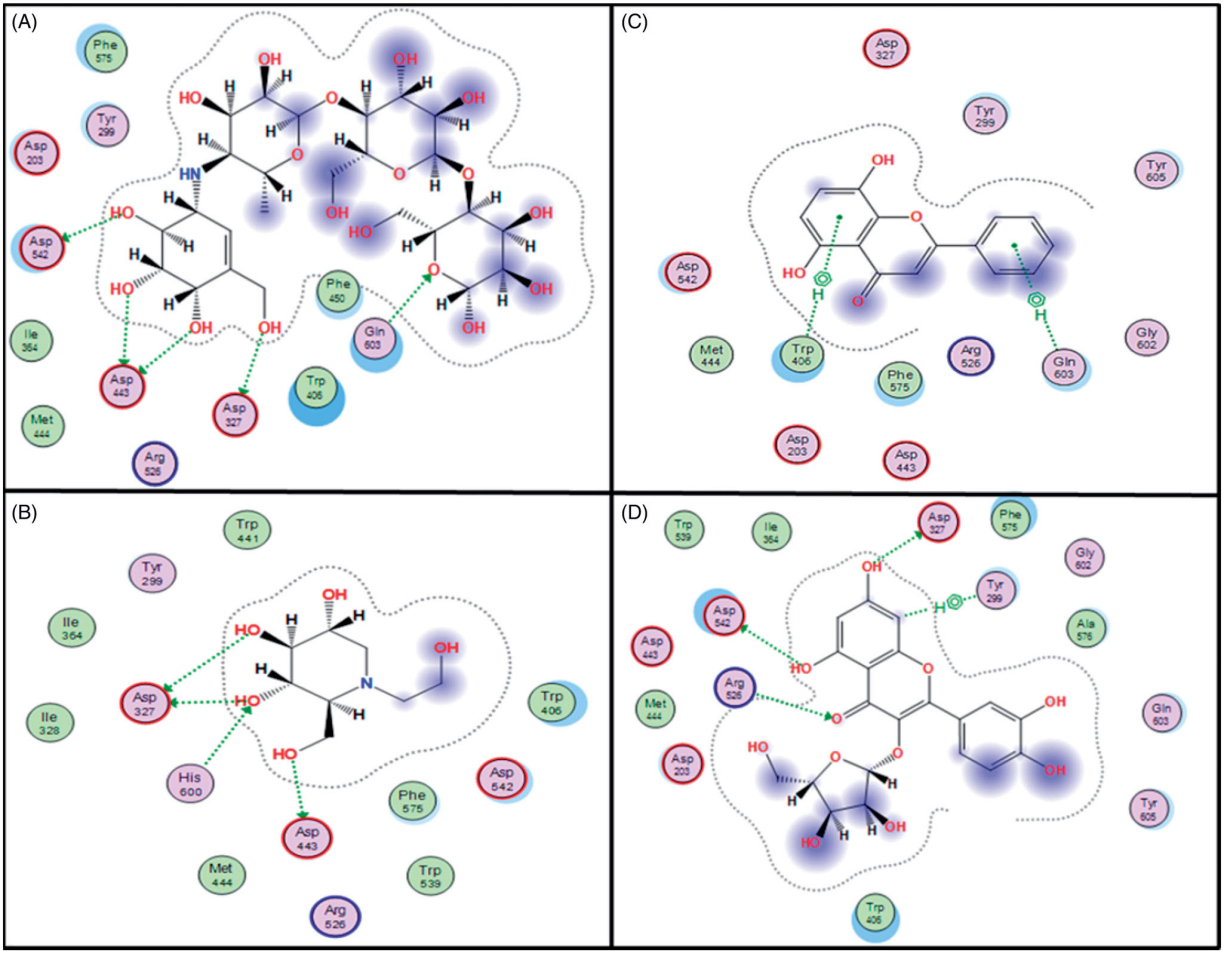
The docking results for selected *Feijoa* constituents compared to acarbose and miglitol in N-terminal of human glucoamylase. 2D representation of enzyme ligand interaction of acrabose (A), miglitol (B), primetin (C) and avicularin (D) in the active pocket of the N-terminal subunit oh human intestinal glucoamylase.

Upon docking in the N-terminal subunit of glucoamylase, the two competitive inhibitors acarbose and miglitol showed multiple H-bond interactions in the active site of the enzyme especially with the acidic residues Asp542, Asp443, Asp327 in addition to Gln603 and His444 (Figure 2(A,B)). Despite being inactive in *in vitro* assay, avicularin showed similar H-bond pattern through its rings A and C with a good docking score of −6.4146 kcal/mol, (Table 3). Other flavonoids showed different mode of interaction which included some H-bond with active site residues (Arg526, Asp542, Asp203) through ring A and/or aromatic stacking with Trp406 (Figure 2). Similar interactions with acidic amino acids Asp1526, Asp1279, Asp1420 in addition to Arg1510 and His1584 were observed upon docking of acarbose and miglitol in the C-terminal subunit. Docking scores for tested compounds in C-terminal subunit were similar to that of N-terminal subunit as seen in Table 3 and ligand–enzyme interactions followed a similar pattern, Figure S2 (provided in the supplementary file). It is worth mentioning that previous molecular interaction data of flavonoids with α-glucosidases were mainly generated using homology model of *Saccharomyces cerevisiae α-*glucosidase enzyme, one of the glycoside hydrolase family 31 which is characterised by presence of aspartate residue in their hydrolytic active site^34^. This is the first report of the molecular interaction of this group of flavonoids in intestinal glucoamylase of the same family showing interaction with the conserved aspartic acid residues in both C and N terminals.

**Table 3.** Docking score of isolated compounds in different target enzymes calculated in kcal/mol.

Samples	AChE	BChE	Amylase	Glucosidase N-terminal	Glucosidase C-terminal	Tyrosinase
*α*-Tocopherol	−9.1515	−8.8952	−7.5147	−6.4146	−6.8471	−6.8136
Flavone	−5.5392	−5.4942	−5.0091	−4.3096	−5.1530	−5.1518
Primetin	−6.2488	−6.0273	−5.1690	−5.0235	−5.4207	−5.4905
Quercetin	−7.1542	−6.0001	−5.5540	−5.1323	−6.1892	−5.7946
Avicularin	−6.6744	−7.0436	−6.6691	−6.4146	−8.0018	−6.7128
*β-*Amyrin	−1.3285	−6.7782	−6.3092	−4.9703	−4.6588	−4.7511

Another clinically significant glucosidase enzyme is the pancreatic α-amylase where upon docking the isolated compounds into its active site, *α*-tocopherol was identified as the most potent ligand with a docking score of −7.514 kcal/mol followed by the glycoside avicularin and *β*-amyrin (Table 3), which interacted *via* H-bonds with Glu233, Asp300, His201 and Arg195 residues in the active pocket similar to the inhibitors; acrabose and miglitol, Figure S3 (provided in the supplementary file). These interactions are also similar to what has been observed with flavonoids interactions in the same enzyme^36^. The fact that interactions of ligand with α-glucosidase and α-amylase active sites are mediated mainly through acidic or basic residues may account for some of the variability observed in *in vitro* assays where differences in pH can influence the assay outcome.

### Anticholineestrases activity

Another debilitating disease especially in elderly population is Alzheimer’s disease (AD). Acetylcholinesterase (AChE) is an established enzyme target for controlling AD conditions by increasing concentration of the neurotransmitter acetylcholine (ACh) in the neurons through the use of AChE inhibitors (AChEIs)^37^^,^^38^. Butyrylcholinesterase (BChE) is another enzyme that hydrolyses acetylcholine and has been shown to be overexpressed in plaques of AD brains possibly as a physiological response to declining concentration of AChE^39^.

*Feijoa* extract and its isolated compounds were investigated for their possible inhibitory activity against both enzymes to explore their potential as treatment for AD in comparison to the standard drug galantamine (GALA). *Feijoa* leaves extract showed good activity against both enzymes estimated at 2.55 and 4.18 mmol GALAE/g of extract against AChE and BChE, respectively, (Table 2).

In general, all chemical constituents of *Feijoa* extract showed good activity against one or both enzymes in comparison to galantamine, a competitive inhibitor for both enzyme^40^. Among tested compounds, primetin was the most active against AChE and BChE at 5.4 and 11.7 mmol GALAE/g, respectively. Flavone, the only flavonoid with no phenolic substitution, showed no activity against AChE while still displayed good inhibitory activity against BChE (10.2 mmol GALAE/g), (Table 2). On the contrary, the most polar of the isolated compounds, avicularin, displayed no activity against BChE and showed the lowest inhibitory activity against AChE in this study at 3.80 mmol GALAE/g. Interestingly, the lipid-soluble α-tocopherol showed good activity against both BChE (10.38 GALAE/g), and AChE (4.92 GALAE/g). This report represents the first report of inhibitory activity of α-tocopherol against AChE and BChE despite several studies that showed a therapeutic benefit of its use in animal models for neurodegenerative diseases^41^^,^^42^. Adding to our interest to this new activity for α-tocopherol is that its lipid solubility can facilitate penetration of blood–brain barrier making it a good drug candidate for neurodegenerative disorders.

**Figure 3.**
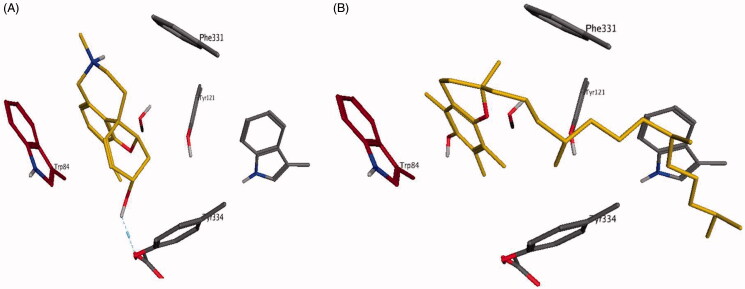
The docking results for α-tocopherol and galantamine in the active site of human AChE. Enzyme ligand interaction of Galantamine (A), α-tocopherol (B) (in gold) in the binding pocket of human acetylcholinesterase where aromatic stacking with Trp86 (in brick red) mediates the binding of galantamine (3.45 Å) with H-bond interaction with Glu202 (2.95 Å) and of α-tocopherol (3.83 Å) and with additional hydrophobic interactions with Phe338, Tyr341, Tyr124, Tyr337 andTrp286.

To further support the previous point, ligand interactions in the active sites of AchE and BChE were studied. First, two commercial inhibitors galantamine and tacrine were docked in the active site of both enzymes to establish the binding mode of these inhibitors. In both enzymes, ligand interactions in the active site were mediated by hydrophobic forces with the indole ring of a key residue Trp86 and Trp82 in AChE and BChE, respectively (Figures 3 and S4). Additional interactions included aromatic stacking with Phe338 and H-bond with Ser203, Glu202 and His447 in AChE and Hist438, Ser79, Asp70 and Thr120 in BChE, which is consistent with previous studies for both enzymes^43^^,^^44^. In support of the *in vitro* results, vitamin E, α-tocopherol, showed the best docking scores at −9.1515 and −8.8952 kcal/mol for AChE and BChE, respectively compared to galantamine at −6.9791 and −6.2397 kcal/mol in the same order. In both enzymes, α-tocopherol was located in the deep binding cavity of the enzyme possibly through strong hydrophobic effect while picking the key interaction with Trp86 in AChE and Trp82 in BChE, respectively (Figures 3 and S4). All other tested compounds were able to interact with either Trp82 or Trp86 except for avicularin which showed different binding mode through its sugar moiety. Also, *β*-amyrin had alternative binding mode for AChE and BChE with no interaction with binding pocket residues. As a result, further investigations of *Feijoa* extract and especially α-tocopherol against these two clinically important enzymes is highly desirable especially in the light of multiple reports of potential protective effects of vitamin E supplementation against dementia in some animal models^42^. Additionally, a synergistic effect of these diverse group of constituents against two enzyme targets with differential expression levels at various stages of AD may add to the potential therapeutic benefit of *Feijoa* extract in AD^39^.

### Antityrosinase activity

Tyrosinase is an essential enzyme in the biosynthesis of melanin and neuromelanin. Anti-tyrosinase compounds such as kojic acid have been used in cosmetic products as skin whitening agent^45^. More recently, overexpression of tyrosinase enzyme in the brain was found to be associated with more production of neuromelanin, a risk factor for Parkinson's disease, PD^46^. Phenolic compounds and flavonoids have been identified as potential inhibitors for tyrosinase enzyme including some flavonoids isolated from different organs of *Feijoa* plant^11^. So, it was worthy to further investigate *Feijoa* leaves extract and some of its isolated compounds for possible anti-tyrosinase activity that have not been investigated before.

The anti-tyrosinase activity of *Feijoa* extract was estimated as 115.85 mmol KAE/g, (Table 2**)**. Among constituents of *Feijoa* extract, quercetin showed the highest activity at 163.05 mmol KAE/g which was supported by previous study by Aoyama and colleagues^11^. In comparison with quercetin, avicularin and primetin showed approximately 45% decrease in activity at 90.85 and 90.06 mmol KAE/g, respectively. It is worth noting that α-tocopherol which is widely used in many skin care and skin whitening products^47^ was reported here to possess good anti-tyrosinase activity at 79.67 mmol KAE/g.

No docking studies were previously performed to explain anti-tyrosinase activity of plant phenolic compounds such as quercetin. Therefore, all isolated compounds were docked in the active site of mushroom tyrosinase along with two known tyrosinase inhibitors; tropolone and kojic acid. The two competitive inhibitors bind to the active site in a similar fashion which included aromatic stacking with Phe264 and Van der Waal interaction with Val283. H-bonds were also observed in both cases with Asn260 and His85 in agreement with published reports^48^. Similarly, flavonoid compounds shared the same pattern while in the case of avicularin, the sugar part (arabinose) added two hydrogen bonds interactions with His85 and Asn260 to result in the best docking score among tested compounds at −6.7128 kcal/mol. Aliphatic derivatives such as *β*-amyrin showed good docking score, (Table 3), but its binding was probably mediated by hydrophobic effects with no specific interactions, Figure S5 (provided in the supplementary file). Generally, it appeared that the inhibitory effect of *Feijoa* extract against tyrosinase enzyme and its potential for therapeutic application is a synergistic effect of multiple components as all tested compounds showed appreciable anti-tyrosinase activity both *in vitro* and *in silico.*

## Conclusion

*Feijoa* plant is attracting renewed scientific attention due to various biological activities exerted by extracts obtained from its different organs^2^. Phytochemical investigations of *Feijoa* leaves extract identified four flavonoids, two steroidal compounds, one triterpene in addition to vitamin E; α-tocopherol. The extract showed excellent antioxidant activity likely due to the presence of powerful antioxidants such as quercetin and *α-*tocopherol.

*In vitro* enzyme inhibition assays and molecular docking studies were used to evaluate biological activity of the isolated compounds. We identified α-tocopherol as a good inhibitor of AChE and BChE through interaction with key amino acid residues in the active site of both enzymes by a combination of H-bonding, π-stacking and hydrophobic forces. Further investigation of the therapeutic benefit of α-tocopherol in AD is strongly recommended given its well-established biosafety profile and good lipid solubility.

Additionally, our investigation offered new insights into some biological activities of the major constituents of *Feijoa* extract. Molecular docking studies were performed to explain anti tyrosinase anti-AChE, anti-BChE of *Feijoa* flavonoids paving the way for further investigating into other flavonoids with different substitution pattern. Also, this study offered the first look into ligand-enzyme interaction of *Feijoa* flavonoids with intestinal glucoamylase. In general, *Feijoa* extract offers a repertoire of bioactive compounds which were demonstrated here through *in vitro* and *in silico* studies to have significant biological activities against drug targets in diabetes, Alzheimer and Parkinson's disease.

## Supplementary Material

Supplemental Material
