# Supplementary material for: Phytochemical profile, enzyme inhibition activity and molecular docking analysis of Feijoa sellowiana O. Berg
Source: J Enzyme Inhib Med Chem. 2021 Feb 8;36(1):618–26. doi: 10.1080/14756366.2021.1880397 (PMC8759727; doi:10.1080/14756366.2021.1880397)
Supplement: Supplemental Material [file IENZ_A_1880397_SM7133.pdf]

In vitro antioxidant properties and key enzymatic drug target of crude extract and isolated compounds from Pineapple Guava - _Feijoa sellowiana_ (O.Berg) O.Berg

.

Table S1: Data for enzymes used in the molecular docking study

Enzyme	PDB code	Ligand	Redocking		Known inhibitor	
			RMSD (Å)	Score	Compound	Score
α-glucosidase	2QMJ	Acarbose	1.3394	-7.1788	Miligitol	-4.8461
α-amylase	1B2Y	Acrabose	1.4206	-8.8956	Miligitol	-5.7597
AChE	4EY6	Galantamine	0.8614	-6.9791	Tacrine	-5.8150
BChE	4BDS	Tacrine	1.2484	-5.4190	Galantamine	-6.2397
Tyrosinase	1Y9X	Tropolone	0.9697	-4.6701	Kojic acid	-5.2330

Docking scores are calculated as kcal/mol

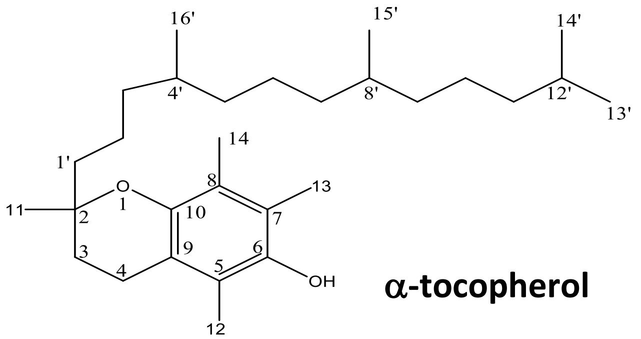

α-tocopherol

Table S2: NMR data for α-tocopherol isolated from *F. sellowiana* leaves extract

	H	C	HMBC
**2**	-	75.6	
**3**	1.7 ,1.6	32.1	118.5, 75.6, 39.9, 23.6, 20.8
**4**	2.48, *t, 6.4*	20.8	146, 118.5, 117.7, 75.6, 32.1
**5**	-	117.7	
**6**	-	144.9	
**7**	-	122.9	
**8**	-	121.1	
**9**	-	118,5	
**10**	-	146.7	
**11**	1.1, *s*	23.6	75.6, 39.9, 32.1
**12**	1.99, *s*	11.8	117.7, 146.7
**13**	2.04, *s*	12.3	144.9 ,122.9
**14**	1.98, *s*	11.3	144.9, 122.9
**1'**	1.4, *m*	39.9	75.6, 39.3, 23.6
**2'**	1.04,1.15, *m*	24.9	39.4,39.9
**3'**	1.01, *m*	39.4	24.9, 37.7
**4'**	1.26, *m*	33.1	19.9, 23.8, 37.7
**5'**	1.13, *m*	37.7	19.9, 37.7
**6'**	0.97, *m*	37.7	33.1, 37.7
**7'**	1.13, *m*	37.7	19.9, 37.7
**8'**	1.26, *m*	33.1	19.9, 37.7
**9'**	1.13, *m*	37.7	19.9, 37.7
**10'**	0.97, *m*	37.7	33.1,37.7
**11'**	1.13, *m*	37.7	28.1,37.7
**12'**	1.4, *m*	28.1	22.9, 37.7
**13'**	0.75,*d,6*.2	22.9	22.9, 28.1
**14'**	0.75, *d,6*.2	22.9	22.9, 28.1
**15'**	0.72, *d,6*.2	19.9	37.7, 33.1
**16'**	0.72, *d,6*.2	19.9	37.7, 33.1

Table S3: NMR data for flavonoids isolated from *F. sellowiana* leaves extract

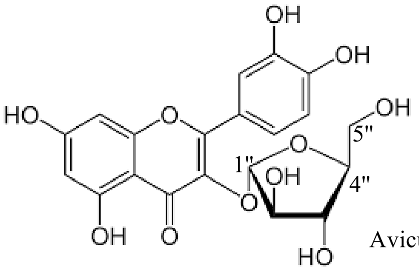

Avicularin

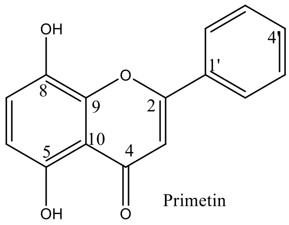

Primetin

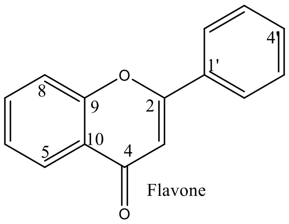

Flavone

	Primetin		Flavone		Avicularin	
**1**	**H**	**C**	**H**	**C**	**H**	**C**
**2**	-	165	-	164	-	157
**3**	6.68 s	106	6.8, *s*	107	-	133.5
**4**		184		178	-	178.6
**5**		161	8.25, *dd,1.7, 8*	125.7	-	161.7
**6**	6.75,*d, 8.4*	110	7.54, *m*	125.3	6.15, *br s*	98.6
**7**	6.91,*d, 8.4*	111	7.72 *dt, 8.4, 2.2*	133.5	-	164
**8**		135	7.59 *d, 8*	118	6.36, *br s*	93.4
**9**		156		157	-	158
**10**		107		124	-	104
**1'**		131		133.6	-	122
**2'**	7.86, *dd 8, 1.8*	127	7.95,*dd, 7.6, 2.2*	126	7.43,*d,1.8*	116
**3'**	7.48, t,8	129	7.55, *m*	129	-	145
**4'**	7.48, t,8	132	7.44 *t, 8*	132	-	149
**5'**	7.48, t,8	129	7.55, *m*	129	6.8, *d,8.3*	115
**6'**	7.86, *dd 8, 1.8*	127	7.95 *dd, 7.6, 2.2*	126	7.4,*dd, 1.8, 8.3*	121.5
**1''**					5.37, *br s*	109
**2''**					4.23,*d,2.8*	77
**3''**					3.4,*m*	82
**4''**					3.4,*m*	86
**5''**					3.75,3.79, *m*	61

Table S4: Antioxidant activity of compounds isolated from *Feijoa sellowiana* leaves' extract.

Samples	DPPH (mmol TE/g)	ABTS (mmol TE/g)	CUPRAC (mmol TE/g)	FRAP (mmol TE/g)	Metal chelating (mmol EDTAE/g)	Phosphomolybdenum assay (mmol TE/g)
α-Tocopherol	90.58±0.89^{c}	80.15±8.55^{d}	81.79±2.35^{d}	55.40±5.57^{d}	0.64±0.04^{f}	1.76±0.20^{c}
Flavone	89.48±0.73^{c}	Na	50.25±0.63^{e}	20.07±0.54^{e}	7.10±0.17^{d}	1.15±0.01^{d}
Primetin	na	Na	16.55±0.16^{i}	10.79±0.52^{g}	3.00±0.82^{ef}	0.88±0.01^{de}
Quercetin	na	1666.46±26.29^{a}	3296.18±69.39^{a}	1989.52±3.48^{a}	31.51±2.11^{a}	6.82±0.47^{a}
Avicularin	994.89±1.23^{a}	166.13±2.57^{b}	432.81±1.06^{b}	138.96±0.65^{b}	4.87±0.17^{de}	1.67±0.10^{c}
β-Amyrin	93.39±0.48^{b}	Na	35.03±0.41^{g}	12.53±2.04^{fg}	10.03±1.05^{c}	0.82±0.05^{def}
β-Sitosterol	Na	Na	24.48±0.81^{h}	12.26±0.17^{g}	1.64±0.30^{f}	0.33±0.04^{f}
β-Sitosterol glucoside	Na	Na	45.42±0.18^{f}	16.48±0.51^{ef}	4.68±0.77^{de}	0.43±0.01^{ef}

Values are expressed as mean±SD. TE: Trolox equivalent. EDTAE: EDTA equivalent.

Different letters indicate significant differences in the samples (p<0.05)

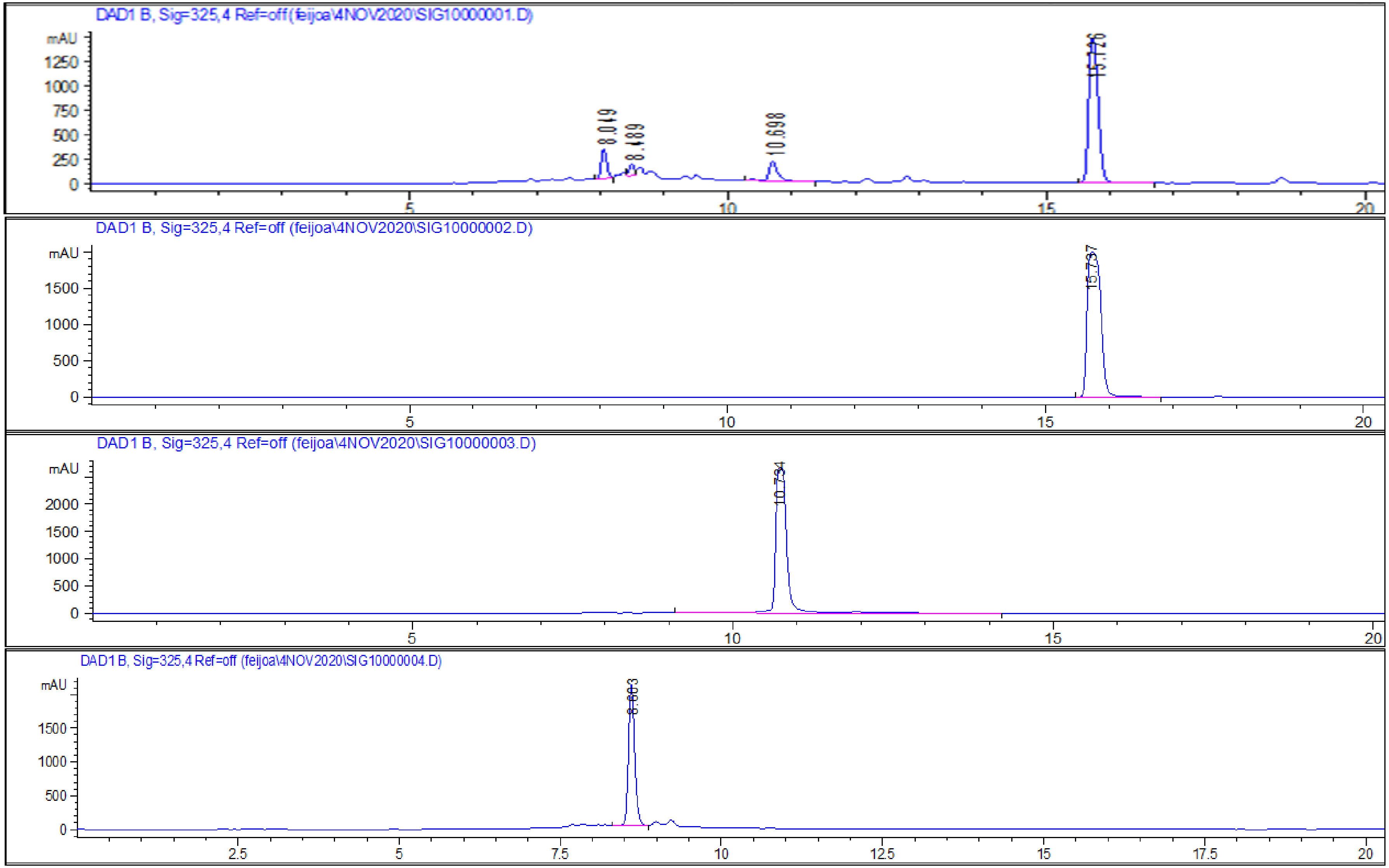
DAD1 B, Sig=325,4 Ref=off (feijoa\4NOV2020\SIG10000001.D)
mAU
8.019
8.489
10.698
15.720
DAD1 B, Sig=325,4 Ref=off (feijoa\4NOV2020\SIG10000002.D)
mAU
15.737
DAD1 B, Sig=325,4 Ref=off (feijoa\4NOV2020\SIG10000003.D)
mAU
10.734
DAD1 B, Sig=325,4 Ref=off (feijoa\4NOV2020\SIG10000004.D)
mAU
8.603

Fig. S1
compo

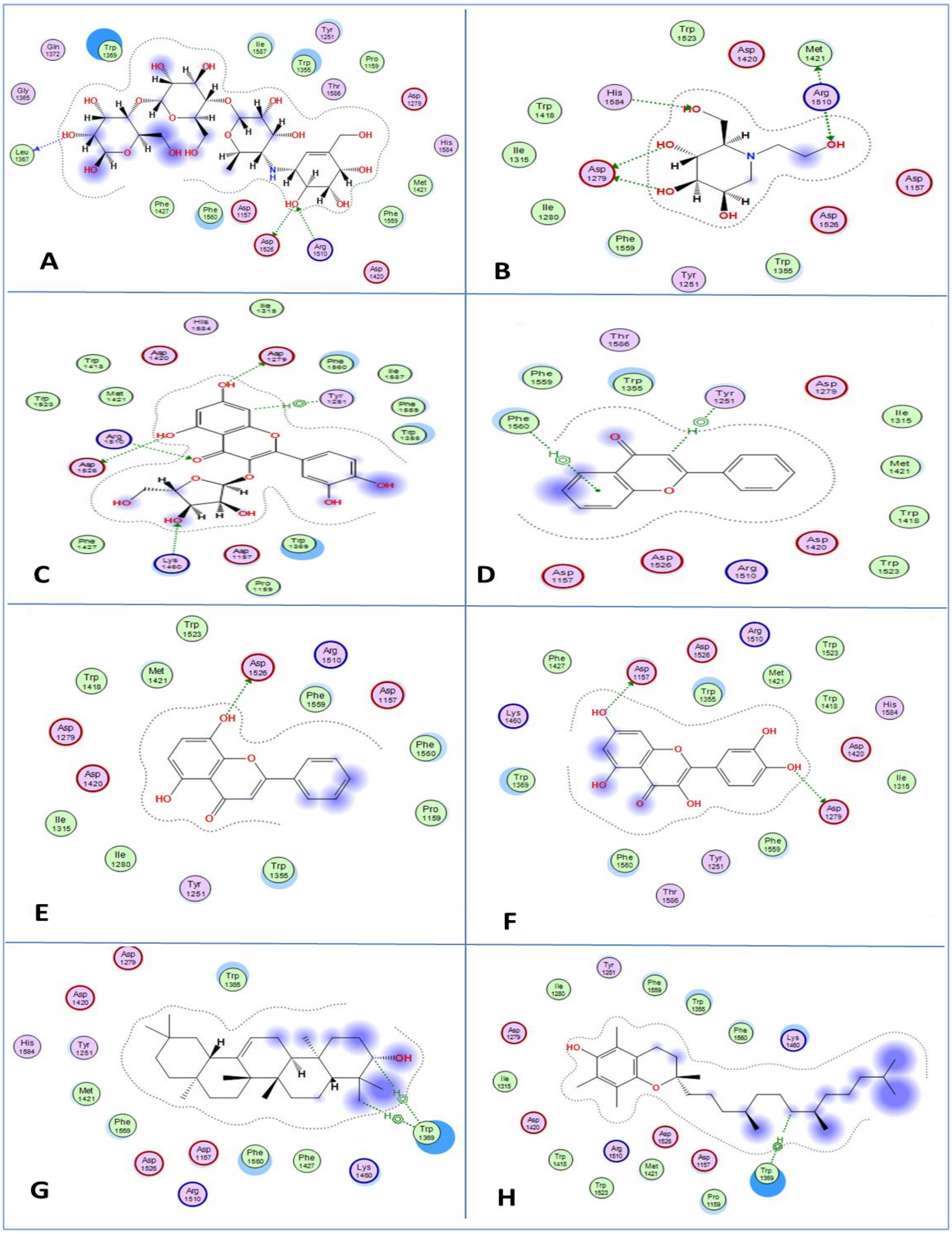

Figure S2: Docking results of *Feijoa* compounds in the active site of C-terminal of human glucoamylase

2D diagram of the enzyme ligand interaction of acarbose (A), miglitol (B), avicularin (C), flavone (D), primetin (E), quercetin (F), β-amyrin (G), α-tocopherol (H)

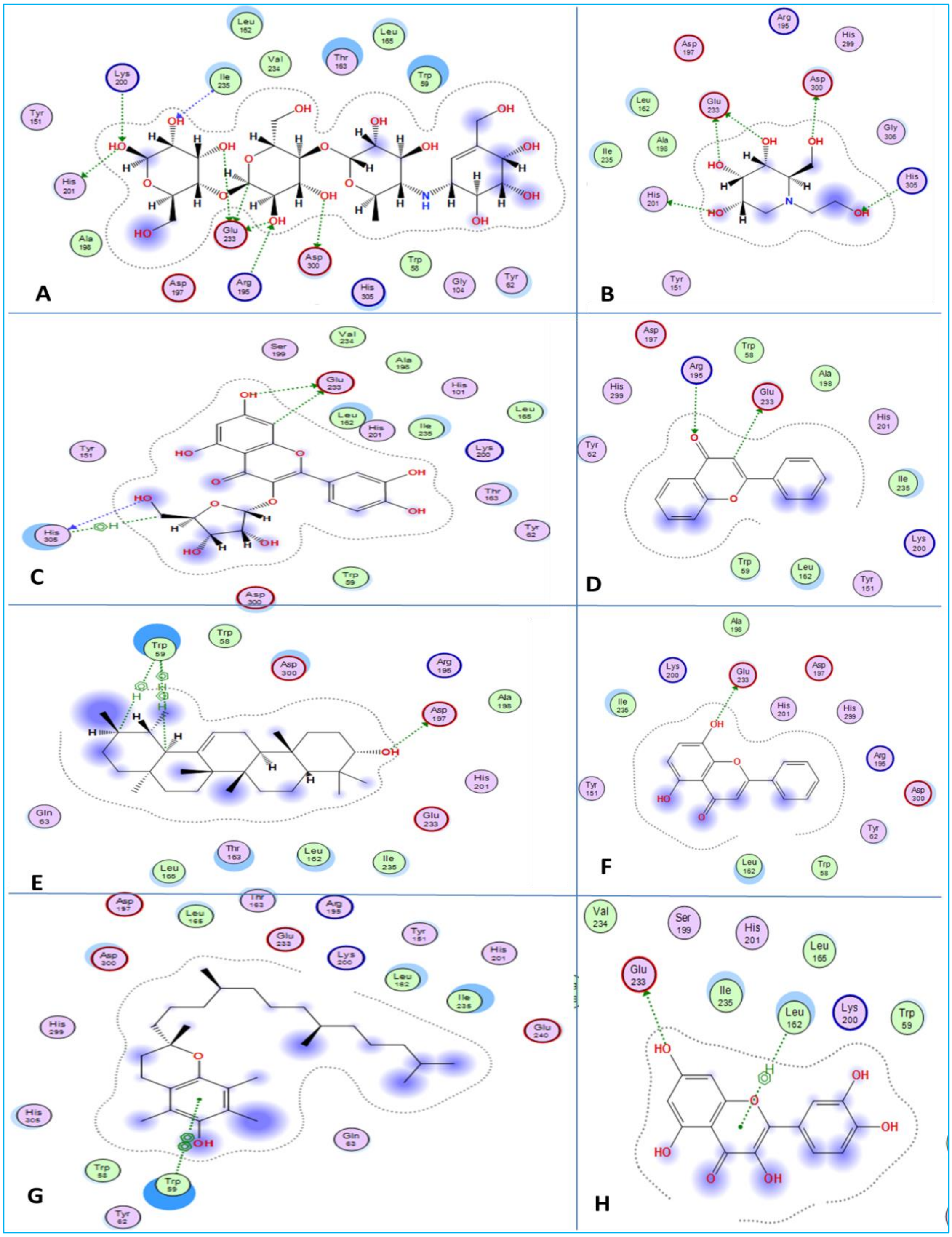

Figure S3: Docking results of *Feijoa* compounds in the active site of human pancreatic α-amylase

2D diagram of the enzyme ligand interaction of acarbose (A), miglitol (B), avicularin (C), flavone (D), β-amyrin (E), primetin (F), α-tocopherol (G), and quercetin (H)

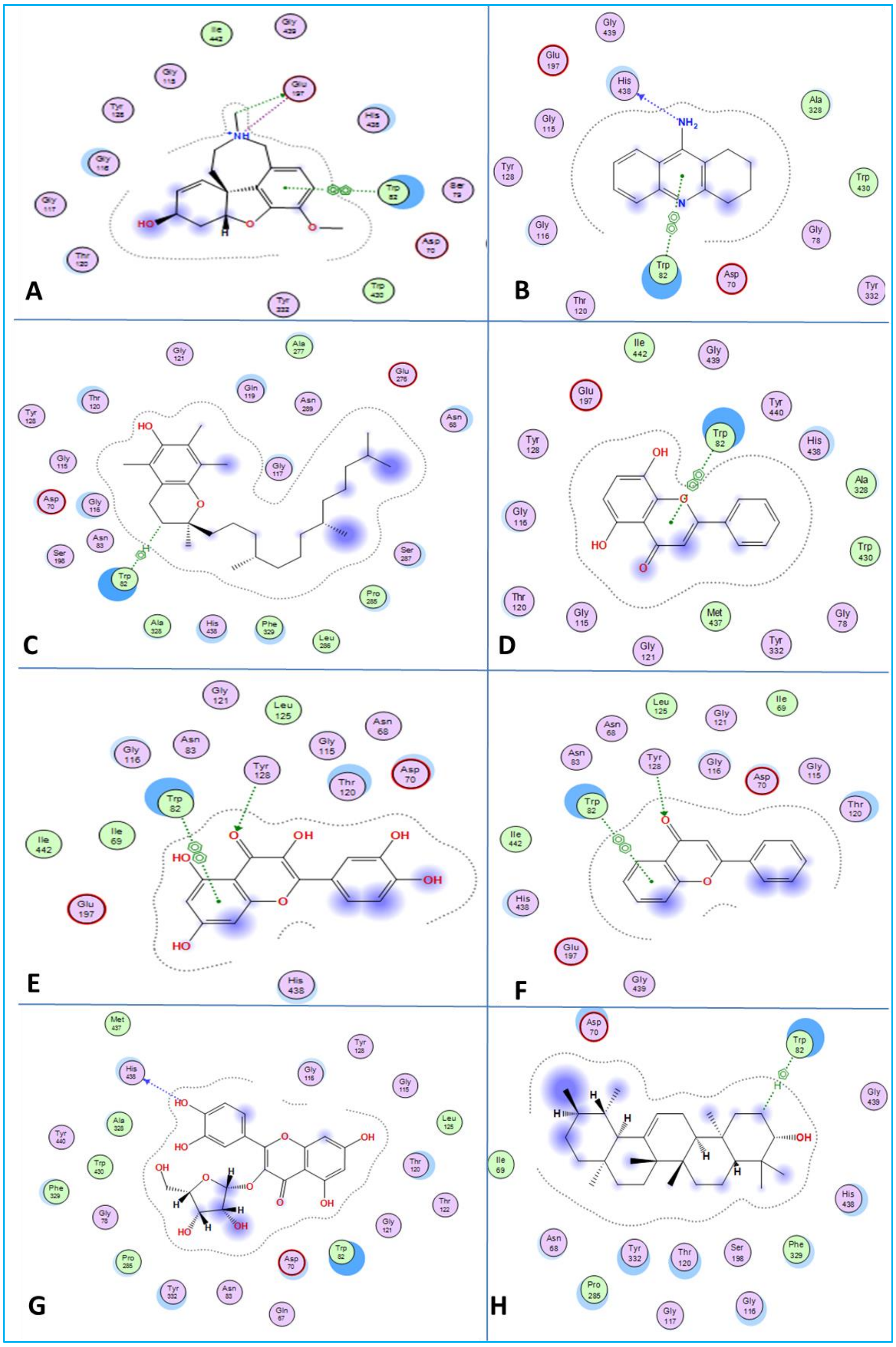

Figure S4: Docking results of *Feijoa* compounds in the active site of human butyrylcholinesterase

2D diagram of the enzyme ligand interaction of galantamine (A), tacrine (B), α-tocopherol (C), primetin (D), quercetin (E), flavone (F), avicularin (G), β-amyrin (H).

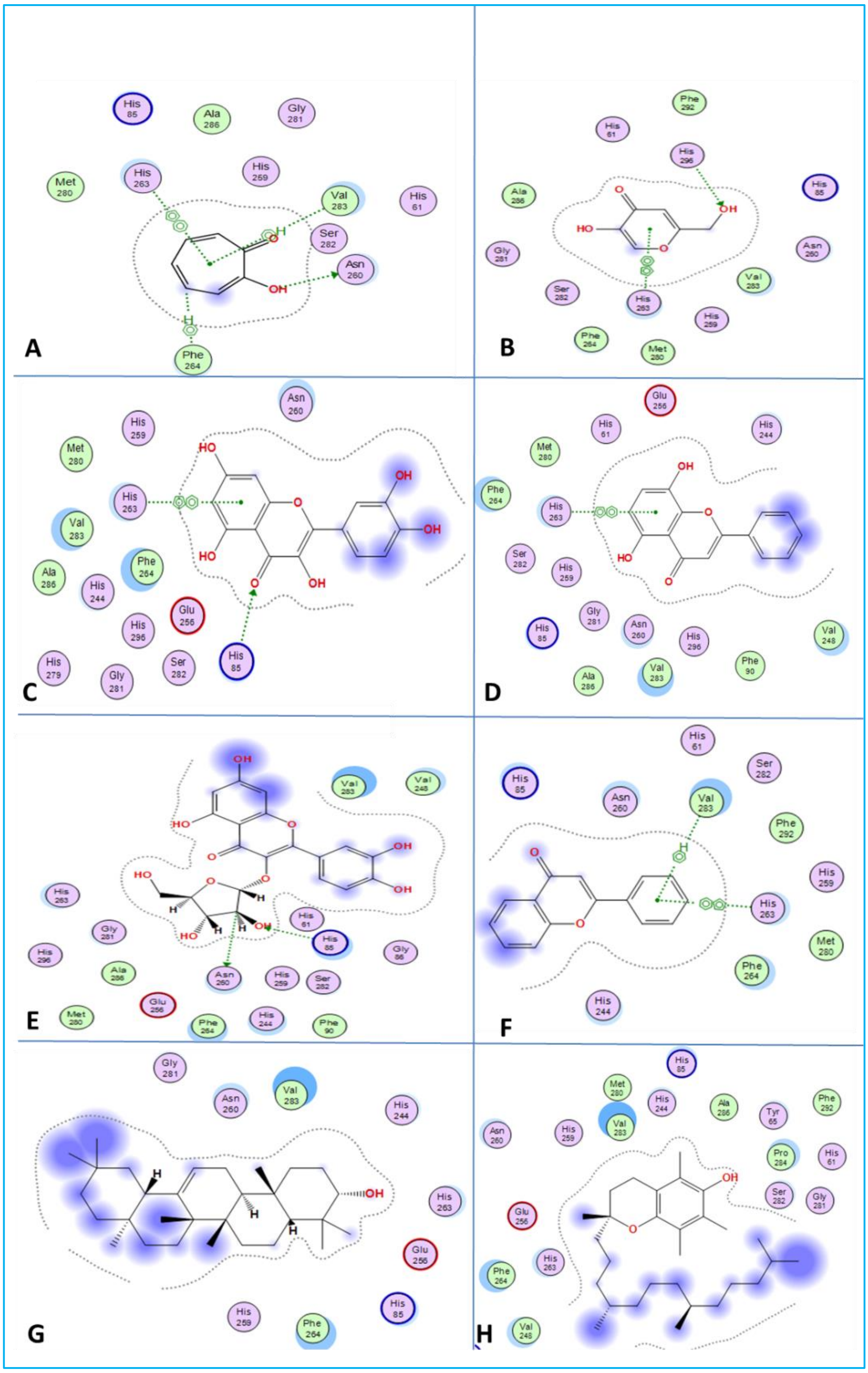

Figure S5: Docking results of *Feijoa* compounds in the active site of mushroom tyrosinase

2D diagram of the enzyme ligand interaction of tropolone (A), kojic acid (B), quercetin (C), primetin (D), avicularin (E), flavone (F), β-amyrin (G), α-tocopherol (H)
